# Genetically Modified Rice: Do Chinese Consumers Support or Go Against It? Based on the Perspectives of Perceived Risk and Trust

**DOI:** 10.3389/fpsyg.2022.813862

**Published:** 2022-08-16

**Authors:** Lingyu Huo, Yan Liu

**Affiliations:** ^1^School of Information, Beijing Wuzi University, Beijing, China; ^2^School of Logistics, Beijing Wuzi University, Beijing, China

**Keywords:** purchase intention, perceived risks, trust, genetically modified rice, health risks

## Abstract

Rice is a staple food in China, and, thus, its security has drawn much attention. The Chinese government proactively fuels the application of biotechnology in agriculture and food to cope with increasingly severe food security issues. However, most consumers resist the commercialization of genetically modified (GM) rice. One of the important reasons is the consumer perception of its various risks. Conversely, trust in the government, scientists, and media can stimulate consumer purchase. On the basis of the dual perspectives of perceived risks and trust, this study establishes a model of purchase intention for GM rice to explore the structural relationship between variables. Perceived risks explore how exclusion can weaken the purchase intention from the consumer perspective; trust examines the benefits that support can provide. Based on the structural equation model, online survey results of 564 consumers in eight provinces and cities are analyzed. The following observations are offered: health risks, moral risks, and purchase intention are negatively correlated; environmental, functional, and economic risks have no significant correlation with purchase intention; and trust and purchase intention have a significant positive correlation.

## Introduction

Rice is an essential food crop and a staple food in the world that feeds more than half of the world’s population and over 60% of Chinese population (Lou et al., 2014; Muthayya et al., 2014; Hu et al., 2018; Shin et al., 2022). China is the largest rice producer and consumer worldwide and devotes approximately 20% of its cultivated area to rice production (OECD, 2004; Chen, 2008; He et al., 2008; Zhang et al., 2022). Food security is the key to the development of the national economy, people’s livelihood, and the national economic security. According to the Food and Agriculture Organization (FAO), food security aims to ensure that everyone can have access to and afford the basic food they need. China is extremely short of food production resources and has always experienced a gap between supply and demand (Fu et al., 2001). Food security in China is considerably challenged. In 1996, the State Council issued the white paper “*The Grain Issues in China*” and put forward the policy of achieving basic food self-sufficiency based on domestic resources, which has become the general outline of the country’s unswerving food strategy. However, China still faces great challenges of fulfilling its promise of “feeding its people.”

The government actively promotes biotechnology in agriculture and food to deal with increasingly severe food security issues in China (Li, 2012). Genetically modified (GM) crops with insect resistance provide unique opportunities to produce improved varieties with insect resistance, thus reducing the loss of grain yield (Chen et al., 2011; Ling et al., 2016; Dastan et al., 2020). Similar to the excessive use of chemical pesticides, wide cultivation of transgenic *Bacillus thuringiensis* (Bt)-resistant crops has allowed them to evolve resistance to Bt genes in rice (Ling et al., 2016). Similar to other GM crops, GM rice is highly promising due to its excellent traits beyond those grown from traditional methods, and has become the key link in the development of modern agriculture in various countries. However, the safety of GM rice has been widely questioned since its inception (Siegrist, 2009; Steur et al., 2012; Zhang et al., 2018). No country or region has approved large-scale commercial planting of GM rice. Although the Chinese government has approved commercial planting licenses for GM cotton, tomatoes, sweet peppers, petunia, poplar, and papaya, only GM cotton has been widely promoted in China. The government has not approved the commercialization of GM crops; even soybeans, corn, and rice are still under field and environmental release tests (Ong and Yang, 2013). Thus, GM technology development has reached a “dilemma”: on the one hand, GM technology can bring economic and social benefits to human beings; on the other hand, unregulated, such technology may cause harm to human health and the environment (Zhang et al., 2018).

In 2009, the GM insect-resistant rice developed by Huazhong Agricultural University won the agricultural GM organism safety certificate issued by the Ministry of Agriculture. However, numerous empirical studies have shown that Chinese consumers are generally opposed to GM rice (Chen, 2008; Zhang et al., 2014b). Moreover, a series of GM rice incidents have occurred in China in recent years, including the “Golden Rice” incident in Hunan Province in 2012, Heinz GM rice cereal incident in 2013, illegal cultivation of GM rice in 2014, sales of GM rice in supermarkets, and the confirmation that batches of rice products from many countries contain GM ingredients. After 10 years of efforts, China still faces a long way to largely commercialize GM rice.

The application of gene technology in rice production can improve yield, improve disease resistance, reduce rice production cost, and enrich its nutritional value (Demont and Stein, 2013; Dobock, 2019). Moreover, the successful commercialization of GM rice will not only help reduce poverty, hunger, and malnutrition worldwide, but also improve the global acceptance of all GM crops. Consumers are the final target in the production and consumption chain of GM crops, and play a key role in food market development (Zhang et al., 2018). Consumers’ willingness can both affect the purchase decision and policy-making for GM rice. Public acceptance of GM rice can both promote and hamper its commercialization and adoption (Amin et al., 2014). In the previous studies, some scholars have focused on the impact of the risks and benefits of GM rice on consumers’ choice (Fu et al., 2001; Li, 2012), while others have focused on the impact of trust (Zhang, 2015; Pang, 2020). However, few researchers pay attention to the simultaneous impact of these two aspects (i.e., risk and trust) on consumers. Thus, this study attempts to establish a model of purchase intention for GM rice based on the dual perspectives of perceived risk and trust to explore the structural relationship between these variables. The negative influence of perceived risk on purchase intention is examined from the perspective of exclusion, while the positive influence of trust on purchase intention is explored from the perspective of support.

## Theoretical Framework and Research Hypotheses

### Theoretical Framework

**Figure 1:**
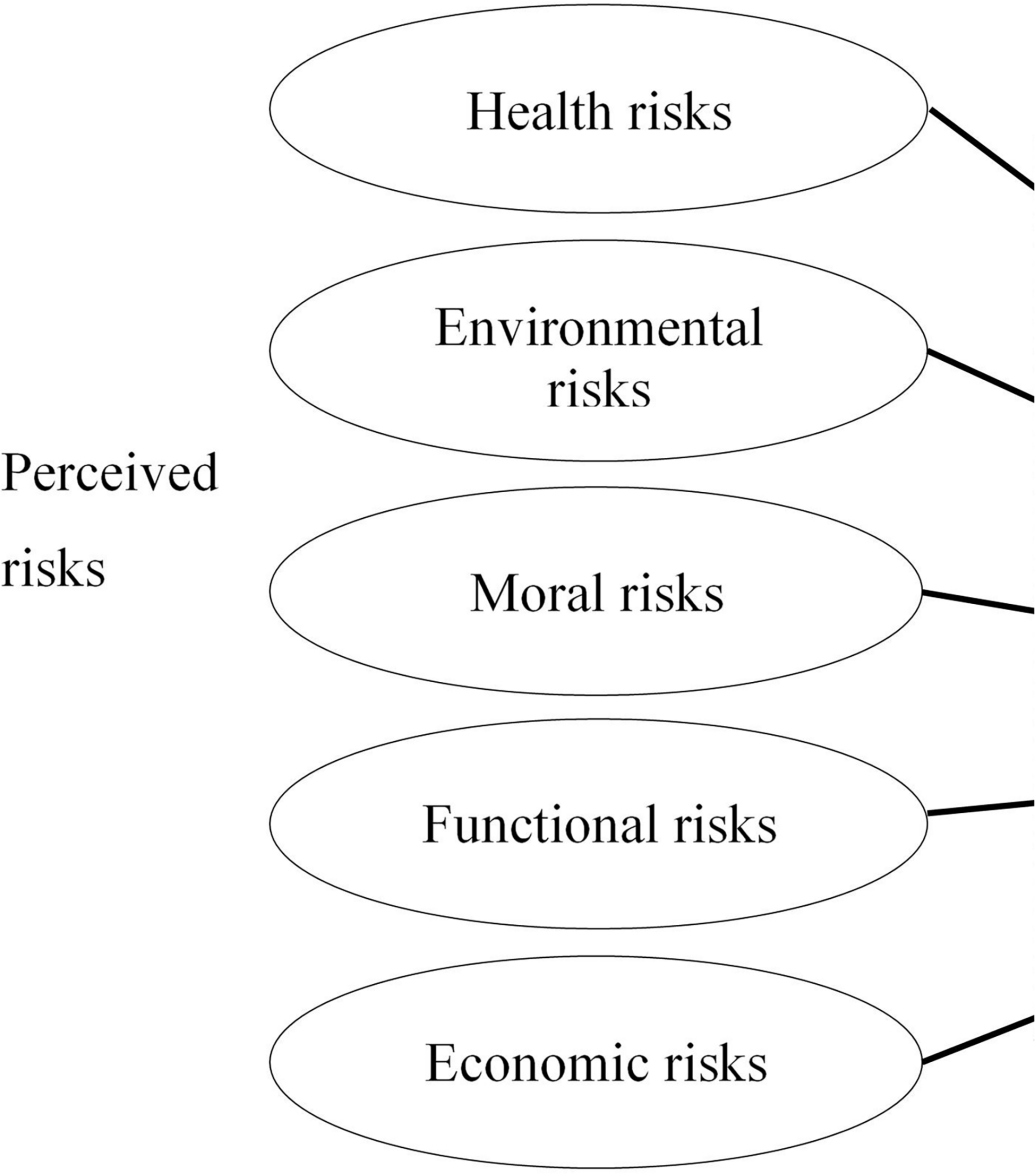
The conceptual model of consumers’ purchase intention toward GM rice and its influencing factors.

Consumers’ purchase intention is simultaneously determined by various factors. BRA (benefits–risks analysis) is a mainstream framework in the research on consumer behaviors (Zhang et al., 2015). According to this model, purchase intention is determined by perceived benefits and risks, which are affected by various factors such as attitude, trust, and knowledge. At the same time, these factors directly or indirectly affect consumers’ purchase intentions. We believe that as rice, a staple food, has a special role in Chinese society, consumers are more sensitive to the perceived risk of GM rice than to its benefit. Therefore, this study mainly explores the effect of perceived risks on purchase intention, a theory that believes risk is an internal experience related to individual psychological, social, and cultural background (Frewer et al., 2004). This concept is first proposed by Bauer, who believed that consumers cannot accurately judge whether the results of their purchase behaviors would be satisfactory (Bauer, 1960). Therefore, consumers’ risk perception affects their purchasing behavior. However, Bauer did not specifically classify perceived risk into different categories. Jacoby and Kaplan (1972) divided perceived risk into five dimensions: financial, functional, social, physical, and psychological aspects. Since then, most of the academic research on consumers’ perceived risks has been carried out from these five dimensions. Peter and Tarpey (1975) added another aspect, “time,” to the five dimensions. Stone and Grønhaug (2013) provided empirical evidence that these six dimensions can be applied to illustrate 88.8% of the total perceived risk. Based on relevant literature (Mao, 2007; Li, 2012; Su, 2013; Yang and An, 2013; Zhan et al., 2013; Jin et al., 2014; Li et al., 2014; Xu, 2014; Zhang et al., 2014a; Xiao, 2015; Chen et al., 2017) and the characteristics of GM rice, we believe that consumers’ perceived risks of GM rice are concentrated in five dimensions, namely health, environmental, moral, functional, and economic risks. In addition, a large number of studies show that consumers’ trust in the government, scientists, and media also affects consumers’ willingness to purchase. On the basis of the theory of perceived risk, Figure 1 defines the meaning of such willingness in a simple conceptual model. Perceived risks and trust are used to assess consumers’ willingness to purchase GM rice from its underlying key factors and perspectives of exclusion and support, respectively.

### Research Hypotheses

There is no evidence of human health effects caused by GM rice. Also, scientific risk assessment never provides a 100% guarantee of there being no risk. Qing and Wu pointed out that health risks are the main reason why Chinese consumers resist GM food (Qing and Wu, 2010). Guo et al. (2013) and Xiao (2019) believed that as consumers prioritize health risks, perceived health risks significantly reduce consumers’ willingness to buy. Therefore, this study proposes the following hypothesis.

*H1*: Consumers’ purchase intention toward GM food decreases according to perceived health risks from GM rice (*H1*).

Jiang (2010) discussed the impact of GM crops on the ecological environment. When GM rice is planted, no competing species may exist in the same ecological environment, which may disrupt the existing ecological balance. Dong et al. (2014) and Xu (2017) proposed that the ecological environment risks perceived by consumers negatively influence the consumers’ purchase intention of GM food. Therefore, the present study proposes the following hypothesis.

*H2*: Consumers’ purchase intention toward GM food decreases according to perceived environmental risks from GM rice (*H2*).

Moon and Balasubramanian (2014) believed that when exploring consumers’ willingness to purchase GM crops, its impact on social morality must be considered. Jiang and Wang (2011) proposed that the reason for the opposition to commercialization of GM rice is born of ethical and moral considerations, such as whether GM rice is “natural” or “unnatural.” Wu et al. (2012) and Xiao (2019) also discovered that the Chinese public is more concerned about the moral risks brought by GM food. Therefore, this study proposes the following hypothesis.

*H3*: Consumers’ purchase intention toward GM food decreases according to perceived moral risks from GM rice (*H3*).

Qing and Wu (2010) showed that functional risks affect consumers’ purchase intention. If the taste and preservation of GM food do not meet the requirements, consumers decline its consumption. Feng (2013) also found that functional risks affect the consumers’ willingness to purchase GM food. Therefore, this study proposes the following hypothesis.

*H4*: Consumers’ purchase intention toward GM food decreases according to perceived functional risks from GM rice (*H4*).

Guo et al. proposed that the health risks mainly determine whether consumers purchase GM food in the first place. Financial risks follow as the second reason (Guo et al., 2013). Xiang et al. (2016) found that consumers’ perceived risks of GM food are mainly concentrated in health, economic, and functional risks: the higher the level of risk perception, the less their willingness to purchase GM rice. Dong (2014) also discovered that economic risks have a significant effect on purchase intention. Therefore, this study proposes the following hypothesis.

*H5*: Consumers’ purchase intention toward GM food decreases according to perceived economic risks from GM rice (*H5*).

Consumers may not be entirely knowledgeable about what the term GM means, nor of the intricacies of different technologies and processes for modifying food (McCluskey et al., 2018). Consumer acceptance of biotech foods will, in large part, be determined by the relative success of the pro-GM and anti-GM groups in influencing public opinion (McFadden et al., 2017). Therefore, consumers often incline to depend on social trust (Earle and Cvetkovich, 1995). Empirical studies show that Chinese consumers relatively lack the knowledge of genetic modification (Huang et al., 2006; Yu and Deng, 2011). To a certain extent, food supply is determined by public products and “decentralized” consumers cannot bear the high cost of information collection and safety supervision of GM food. The vast majority of the public lack relevant professional expertise, and cannot understand and control the risks and uncertainties of GM food by themselves. They considerably rely on expert evaluation of new products to make their judgments (Zhang et al., 2018). Gaskell et al. showed that consumer trust in government, experts, and media reports has a significant impact on risk perception and whether the consumers tend to choose GM crops and food. If consumers trust the government and experts, then their risk perception decreases; if consumers have a low degree of trust, then their risk perception becomes more sensitive (Gaskell et al., 2003). Ortega et al. (2011) and Zhang (2015) also showed that consumers’ trust in government certification programs, third-party certification agencies, and GM labeling systems causes the Chinese consumers much inclined to pay for GM food. Therefore, this study proposes the following hypothesis.

*H6*: Consumers’ purchase intention toward GM rice increases according to consumers’ trust in related institutions (*H6*).

## Materials and Methods

### Questionnaire

The questionnaire consists of four parts. The first part is basic personal information and profile. The second part is perceived risks, including health, environmental, moral, functional, and economic. The third part is trust while the fourth part is purchase intention. A 5-Point Likert Scale is adopted for the questionnaire, with “strongly disagree,” “disagree,” “indifferent,” “relatively agree,” and “strongly agree” corresponding to 1, 2, 3, 4, and 5 points for purchase intention. For trust, the scale adopts “very distrustful,” “not too trusting,” “indifferent,” “relatively trusting,” and “very trusting” for 1, 2, 3, 4, and 5, respectively. The content was designed based on previous works of scholars at home and abroad. The measurement items have passed strict reliability and validity tests in advance. Before the official survey, a pilot study of 50 people is carried. Respondents were asked whether the content in the questionnaire is clear and reasonable; then, based on the results, the unreasonable parts are modified. Table 1 shows the final questionnaire variables and measurement items.

**Table 1:** Variables, measurement items, and sources of the questionnaire.

Variables		Measurement items	Sources
Perceived risks	Health risks	HR1 GM rice may cause long-term harms to our health	Bredahl (2001)
		HR2 GM rice may cause allergic reactions in the human body	
		HR3 Compared with GM rice, traditional rice is healthier	
		HR4 GM rice may harm your health and that of your families	
	Environmental risks	EnR1 GM rice may cause long-term adverse effects on our ecological environment	Bredahl (2001)
		EnR2 GM rice may destroy the ecological balance of nature	
		EnR3 The cultivation of GM rice may harm the environment	
		EnR4 the impact of GM rice on the environment may threaten human survival	
	Moral risks	MR1 GM rice is morally unacceptable	Christoph et al. (2008)
		MR2 GM rice violates the evolutionary laws of nature and life	
		MR3 Importance has been attached to the ethics and integrity of food production enterprises	
		MR4 The commercialization of GM rice may disrupt social and moral order	
	Functional risks	FR1 The more natural the food traits are, the much better it is	Gaskell et al. (2006)
		FR2 GM rice may be featured by unnatural characteristics	
		FR3 GM rice may be inferior to traditional rice in terms of nutrition and taste	
		FR4 Several characteristics of GM rice do not meet your expected requirements	
	Economic risks	EcR1 The commercialization of GM rice may be only in the interests of large food production companies and a few people	Christoph et al. (2008)
		EcR2 I am concerned about whether the food is affordable and economical	
		EcR3 the price of GM rice may be lower than traditional rice	
		EcR4 Having GM rice may bring medical burden to future generations	
Trust		T1 How much trust do you have in the public opinion about GM rice on the Internet?	Chen and Li (2007)
		T2 How much trust do you have in scientists’ reports on GM rice?	
		T3 How much trust do you have in the report of GM rice?	
		T4 How much trust do you have in the government’s supervision and policy reports on GM rice?	
Purchase intention		P1 Possibility of purchasing transgenic rice in the future	Kim et al. (2014)
		P2 If the price of GM rice is cheaper than that of ordinary rice, the possibility of purchase is selected	

GM rice in this questionnaire refers to the transgenic rice with insect resistance.

### Survey Targets

In this study, the online questionnaire^1^ was distributed through WeChat and QQ. From March to May 2018, a total of 612 questionnaires from eight provinces and cities across China (including Shandong Province, Henan Province, Shanxi Province, Hubei Province, Hunan Province, Guizhou Province, Tianjin City, and Hebei Province) were collected. The residents of these eight provinces and cities are selected as samples, mainly for the following reasons: first, the main food of residents in these provinces and cities is basically rice; second, the total resident population of these provinces and cities accounts for nearly 40% of China’s total population, which is fairly representative. During the survey, to mobilize the respondents, we distributed a certain amount of “red envelopes” to those who submitted the questionnaire. At the same time, to avoid duplication of data, we determine that each IP address can only submit one questionnaire. A review of the 612 questionnaires showed that 48 questionnaires could not meet the requirements and were eliminated. Finally, 564 valid questionnaires were obtained, with a validity rate of 92.1%. Table 2 shows the descriptive statistics of the samples used in this study, including gender, age, education background, and places of residence.

**Table 2:** Descriptive statistics of the sample data.

Demographics	Item	Number	Sample (%)	Population (%)	$\chi^{2}$ tests
Gender	Male	287	50.9	51.3	0.21
	Female	277	49.1	48.7	
Age	18–30	260	46.0	37.8	5.73
	31–40	122	21.6	23.7	
	41–50	83	14.7	23.7	
	51–60	90	16.0	14.8	
Place of residence	Rural	199	35.28	50.3	1.08
	Urban	132	64.72	49.7	
Education level	Junior high school graduation	25	4.43		
	High school graduation	60	10.64		
	Bachelor/Junior college graduation	423	75.00		
	Master degree or above	56	9.93		

A *χ*^2^ test was applied to ensure that the sample in this study are representative of the entire population. Table 2 presents the characteristics of the sample and the *χ*^2^ test results, which indicate that the sample could represent the Chinese population (*p* < 0.05).

### Structural Equation Model

Structural equation model (SEM) is a statistical method that uses a system of linear equations to express the relationship between observed and latent variables or the relationship between latent variables. This model has been increasingly applied to the fields of management, psychology, sociology, and other social sciences (Lin and Jiang, 2006; Hair et al., 2012). Various latent variables involved in social and psychological research cannot be accurately and directly measured. SEMs can be used to study the relationship between these latent variables and their indicators by setting observed variables (Hou et al., 2004). This study uses several observed variables to study the relationship between perceived risks (including health, environmental, moral, functional, and economic risks), trust, and purchase intention. Among these, various perceived risks and trust are used as exogenous latent variables, while the willingness to purchase is regarded as an endogenous latent variable.

## Results

### Measurement Model

Through principal component analysis, the variables HR3, MR3, FR1, EcR2, and EcR3 fail the test and are thus deleted. Then, the deleted and sorted data are imported into Amos 20.0. The fitness index of the measurement model is obtained as follows: Chi-square/degrees of freedom = 2.192 < 3, *p* < 0.001, indicating that the results are significantly different, GFI = 0.945, AGFI = 0.918, NFI = 0.924, IFI = 0.957, CFI = 0.956, and RMSEA = 0.051 < 0.08. These data show that the fitting indexes of the measurement model meet the requirements.

Cronbach’s α coefficient is used to test the reliability of the measurement items. The results show that the Cronbach’s α value of each variable is greater than 0.5, which indicates that the reliability of each variable is acceptable. Next, we carried out loading, average variance extracted (AVE), and combined reliability (CR) evaluation. Table 3 shows the results. The empirical rule proposed by Carmines and Zeller stated that the load value greater than 0.70 is acceptable (Carmines and Zeller, 1979), while Bagozzi and Yi believed that a load value greater than 0.50 is acceptable (Bagozzi and Yi, 1988). For the filtered samples of variables H3, V3, F1, M2, and M3, the combined reliability of all variables is greater than 0.7, which is highly consistent. In addition, the AVE values of the samples in the model are all greater than 0.50, which meets the standard value suggested by Fornell and Larcker (1981). These results indicate that the measurement model has sufficient load, reliability, and consistency.

**Table 3:** Measurement model and data.

Variables	Indicators	Loading	CR	AVE
Health risks	HR1	0.766	0.751	0.502
	HR2	0.682		
	HR4	0.675		
Environmental risks	EnR1	0.728	0.852	0.591
	EnR 2	0.782		
	EnR 3	0.818		
	EnR 4	0.743		
Moral risks	MR1	0.742	0.855	0.664
	MR2	0.775		
	MR4	0.917		
Functional risks	FR2	0.781	0.759	0.515
	FR3	0.610		
	FR4	0.751		
Economic risks	EcR1	0.738	0.735	0.581
	EcR 4	0.786		
Trust	T1	0.512	0.850	0.596
	T2	0.760		
	T3	0.839		
	T4	0.916		
Purchase intention	P1	0.778	0.757	0.609
	P2	0.783		

**Figure 2:**
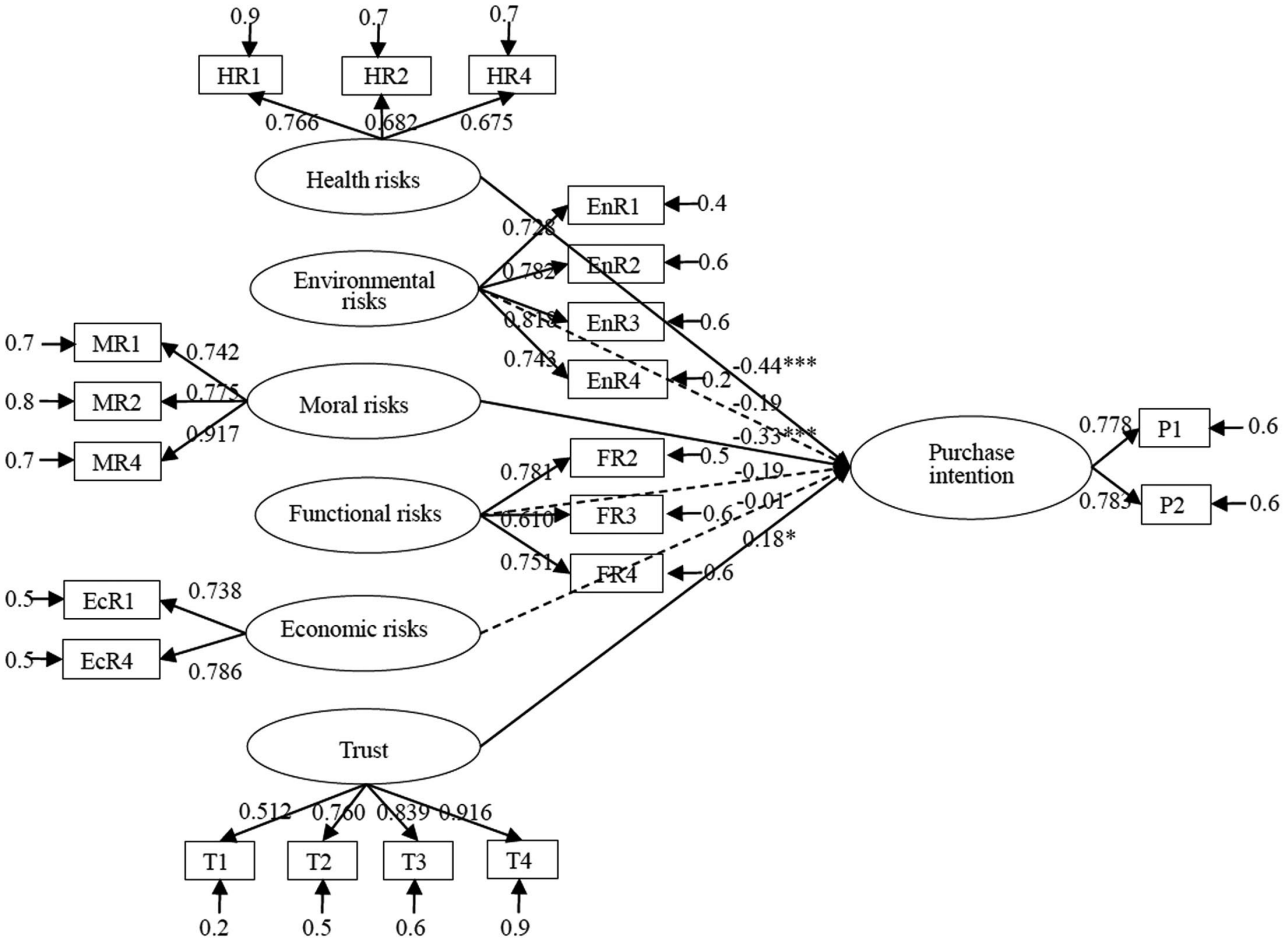
Structural equation model of consumers’ willingness to purchase GM rice.

Based on the above data analysis, Figure 2 illustrates various types of perceived risks (including health, environmental, moral, functional, and economic risks), trust, and purchase intentions among model outputs and path coefficients with the use of Amos 20.0 software.

### Structural Model

In this study, perceived risks and trust are taken as exogenous latent variables and purchase intention is an endogenous latent variable. Table 4 shows the path coefficient and *t* value, significance level, and hypothesis test results.

**Table 4:** Path coefficient and hypothesis test of structural model.

Path	H	Coefficient	*t*	Sig.	Results
Health risks → Purchase intention	H1	−0.435	−3.059	^***^	Supported
Environmental risks → Purchase intention	H2	−0.187	−1.785	0.074	Not supported
Moral risks → Purchase intention	H3	−0.327	−3.195	^***^	Supported
Functional risks → Purchase intention	H4	−0.189	−1.751	0.080	Not supported
Economic risks → Purchase intention	H5	−0.008	−0.111	0.912	Not supported
Trust → Purchase intention	H6	0.179	2.287	^*^	Supported

* *p < 0.05;*

*** *p* < 0.001.

Health risks have a significant effect on purchase intention and its standardized path coefficient is −0.435 (*p* < 0.001). This finding shows that the higher the health risk perceived by consumers, the less they are willing to purchase. Therefore, Hypothesis 1 is supported. Environmental, functional, and economic risks have standardized path coefficients of −0.187, −0.189, and − 0.008 to purchase intention, respectively, and *t* values of −1.785, −1.751, and − 0.111, respectively. None of these values pass the examination. This finding shows that environmental, functional, and economic risks have no statistically significant correlation with purchase intention. Therefore, Hypotheses 2, 4, and 5 are not supported. Moral risks have a significant impact on purchase intention. Their standardized path coefficient is −0.327 and the significance level is 0.001, indicating a significant negative correlation. Therefore, Hypothesis 3 is supported. The standardized path coefficient of trust to purchase intention is −0.179, *p* < 0.05, indicating a significant positive correlation between trust and purchase intention. This shows that as consumers’ trust in related institutions increases, they become more inclined to purchase GM rice. Therefore, Hypothesis 6 is supported.

## Discussion

Research on the purchase intention of GM rice and exploration of the impact of related factors on consumers is of great significance to the development of this technology in China (Zheng, 2015). As a major agricultural producer and consumer, China is greatly challenged by the long-term effective supply of major agricultural products. Similar to many other developing countries, China has to decide how to develop and commercialize GM technology. This study is based on the survey of 564 consumers in eight provinces and cities from March to May 2018. SEM is used to examine how the two major influencing factors, namely, perceived risks (including health, environmental, moral, functional, and economic risks) and trust affect the purchase intention from the dual perspectives of exclusion and support. The main conclusions are as follows: first, a significant negative correlation exists between health and moral risks, and purchase intention; second, no significant correlation exists between environmental, functional, and economic risks, and purchase intention; and third, a significant positive correlation exists between trust and purchase intention.

Perceived benefits and risks are regarded as important predictors for the purchase intention of GM food (Chen and Li, 2007; Zhang et al., 2018). Rice is the staple food of the Chinese, and, thus, consumers are relatively cautious about buying GM rice. We believe that perceived risks may have a greater effect on consumers’ purchase intentions compared with its perceived benefits. Therefore, this study focuses on the impact of perceived risks on purchasing intentions of GM rice, which appear to be many, such as health, environmental, moral, functional, and economic risks. However, empirical results show that various risks can actually affect consumer willingness. To this end, to “change” consumers’ “prejudice” toward GM rice, we can start with the health and moral risks, which are the main consumers concern. Through a reasonable risk communication mechanism, consumers can correctly and scientifically recognize health and Moral risks. Of course, this result may be also partly due to our survey sample (18–30 age group accounts for 46%, of whom 75% have undergraduate/college degree are). Compared with other social groups, young consumers are more open and innovative, and their attitude toward genetic modification is more optimistic (Chen et al., 2017). In addition, knowledge and education are proposed to have a significant effect on the acceptance and purchase intention of GM food (Moerbeek and Casimir, 2010; Vilellavila et al., 2010; Rodríguezentrena and Salazarordóñez, 2013).

Consumers mainly worry that GM rice may harm their health and their families. In terms of the health risks of GM rice, currently, no safety guarantee exists at home and abroad, and most consumers do not fully understand whether GM rice is safe or not. As health should be prioritized and the right to health should not be violated, the possible damage and negative effects of GM rice on human health cannot be ignored. In the case of uncertain safety, GM rice cannot be defined as a healthy food without causing problems (Zhang and Liu, 2012). Current scientific evidence shows that genetic methods used for specific crop improvement do not harm the health of humans and animals (Chen and Chen, 2016). However, the safety of GM crops has not been tested for a long period of time (hundreds of years). In this case, the government should guide consumers to correctly and reasonably understand the possible health risks of GM rice and assume a cautious approach to its commercialization. Fortunately, relevant departments and several experts have realized the importance of information transparency and popular science. In addition, systematic response to the safety issues and health risks is enacted and biosafety management procedures, processes, and applications involved in GM technology, which are of general concern to the public, are adopted (Li, 2012). Understanding the issues of public concern and their integration is very important for policy-making (Zhang et al., 2018).

The present results show that the more consumers trust related institutions and information sources, the stronger they become willing to purchase GM rice. The Chinese government used to emphasize the research, promotion, and safety management of GM technology. In fact, the government should pay more attention to the popularization of GM knowledge to promote the industrialization of GM food. Enhancing the popularization of GM knowledge can improve the public awareness of GM food, help the public better understand the nature of GM technology, and eliminate doubts about GM food. The survey results show that, in terms of Chinese consumers’ trust in scientific research institutions, scientists rank first place followed by the governments, manufacturers, and networks. Therefore, scientists and governments should play a more active role in spreading the benefits of GM food to the public. At present, due to loopholes in the supervision of GM rice, illegal cultivation and transaction of GM rice in the market has occurred several times. Therefore, consumers start to panic and resist GM rice. Even worse, they become inclined not to trust the scientific research institutions, food companies, and the government. The government should strengthen supervision and increase public trust, and ensure that the GM rice sold in the market aims to improve the overall welfare of consumers. Without clarification of its risks, GM rice should not be circulated in the market through illegal channels and further harm consumers’ confidence in the government, scientific research institutions, and production enterprises.

GM rice has always faced controversy, including the first assessment of its commercialization by the Ministry of Agriculture in 2004, the safety certificate for the production and application of GM rice issued by the Ministry of Agriculture in 2009, and a series of GM rice incidents in recent years. The focus of the debate is “what are the risks of GM rice.” Scientists are more supportive of GM rice. However, several scholars, non-governmental organizations, the media, and the public are more opposed to GM rice (Lin and Jiang, 2006). Over the past decade, national policies seem to have undergone “subtle” changes toward GM technology and its commercialization. From 2007 to 2018, the Central Document No. 1 mentioned GM technology and its application seven times, as follows: “strictly implementing the GM food labeling system” in 2007; “accelerate research and accelerate commercialization” in 2009–2010; “strengthen the popularization of GM science” in 2015; and “prudent promotion on the basis of ensuring safety” in 2016. The “subtle” changes in policies are highly related with consumers’ perception and acceptance of GM food. For example, in October 2014, the Ministry of Agriculture responded to the suspension of the importation of a certain GM soybean with the main reason of “low public acceptance.”

Is the future of commercialization of GM rice pessimistic? Based on the survey results of this study, consumers’ willingness to purchase GM rice is much higher than expected. Thus, as the government strengthens the supervision of GM technology and its application, scientific and technological communities continuously explore GM technology, government and scientists continuously popularize GM knowledge, and the business community continuously improves the research and development of GM technology, Chinese consumers becomes more willing to purchase GM rice. Therefore, the commercialization of GM rice remains promising (Mao, 2011).

## Conclusion

On the basis of the dual perspectives of perceived risks and trust, this study establishes a model of purchase intention for GM rice, a staple food in China, to explore the structural relationships between variables. Compared with environmental risk, functional risk, and economic risk, health risk and moral risk have stronger impacts on the purchase intention of Chinese consumers; trust and purchase intention have a significant positive relationship. Therefore, the results presented in this study are useful for understanding the effect of risk and trust variables on purchase intention of Chinese consumers toward GM rice. Several methodological limitations of the present study should be acknowledged. First, the sample was selected only from eight provinces and cities in China. Second, the distribution of education level in the sample is too concentrated, in which the number of Bachelor/Junior college graduation accounts for 75%, which may have an impact on the overall results. Finally, the relationship between risk and trust should be considered for further studies.

## Data Availability Statement

The original contributions presented in the study are included in the article/supplementary material, further inquiries can be directed to the corresponding author.

## Ethics Statement

Ethical review and approval was not required for the study on human participants in accordance with the local legislation and institutional requirements. The patients/participants provided their written informed consent to participate in this study.

## Author Contributions

LH: writing original draft, conceptualization, and reviewing draft. YL: data collection and data analysis. All authors contributed to the article and approved the submitted version.

## Funding

This study is funded by Beijing Great Wall Scholars Program (Grant no. CIT&TCD20190320).

## Conflict of Interest

The authors declare that the research was conducted in the absence of any commercial or financial relationships that could be construed as a potential conflict of interest.

## Publisher’s Note

All claims expressed in this article are solely those of the authors and do not necessarily represent those of their affiliated organizations, or those of the publisher, the editors and the reviewers. Any product that may be evaluated in this article, or claim that may be made by its manufacturer, is not guaranteed or endorsed by the publisher.

## Footnotes

^1^ www.wjx.cn
